# Research on Measurement of Coal–Water Slurry Solid–Liquid Two-Phase Flow Based on a Coriolis Flow Meter and a Neural Network

**DOI:** 10.3390/s25113267

**Published:** 2025-05-22

**Authors:** Jie Liu, Lingfei Kong, Jiahao Ma, Xuemei Zhang, Chengjie Wang, Dongze Wu

**Affiliations:** 1 School of Mechanical and Precision Instrument Engineering, Xi’an University of Technology, Xi’an 710048, China; 1240210004@stu.xaut.edu.cn (J.M.); 15129184114@163.com (D.W.); 2 College of Chemistry and Chemical Engineering, Yan’an University, Yan’an 716000, China; zxmysmm@126.com (X.Z.); 19891105139@163.com (C.W.)

**Keywords:** coal–water slurry (CWS), error, deep learning

## Abstract

The development of coal–water slurry (CWS), a new type of coal-based chemical product in China, has garnered increasing attention as a potential substitute for petroleum resources. The Coriolis mass flow meter is widely used in industrial measurement due to its low uncertainty and its ability to simultaneously measure fluid density and mass flow rate, with a single-phase measurement error as low as 0.1%. However, significant errors still exist in multiphase flow measurement scenarios. To address this issue, we designed and constructed a CWS liquid–solid two-phase flow measurement platform to investigate the flow measurement errors of CWS in Coriolis mass flow meters under various conditions. A deep learning correction framework was developed to mitigate the significant measurement errors in liquid–solid two-phase flow. Based on the theoretical support provided by repeatability experiments, two correction models were established: (1) An error correction model based on a BP neural network was developed, which provided corrections for the measurement errors of CWS liquid–solid two-phase flow. The first correction results showed that the corrected error of the predictive model was 3.98%, a significant improvement compared to the 5.11% error measured by the X company’s meter. (2) Building on this, a second correction model was established through algorithm optimization, successfully reducing the corrected error of the predictive model to 1.01%. Through this study, we aim at providing a new technical approach for Coriolis mass flow meters in the field of liquid–solid two-phase flow measurement, enhancing measurement accuracy, reducing costs, and offering more reliable data support for industrial process control and scientific research.

## 1. Introduction

In most parts of the world, the energy production framework relies heavily on fossil fuels. According to the 2019 World Energy Outlook, approximately 64% of electricity is produced using fossil fuels, with coal alone accounting for 38% of total electricity generation [1]. China’s energy structure is dominated by coal (accounting for over 70%), accompanied by a shortage of petroleum resources and dependence on imports. CWS, as a clean coal-based fuel, can directly replace fuel oil (2 tons of CWS ≈ 1 ton of fuel oil), significantly reducing dependence on petroleum and ensuring energy security. Its promotion is in line with the national strategy of “replacing oil with coal”, especially in the fields of industrial boilers, coal chemical industry, etc., exhibiting important economic value.

CWS displays a high combustion efficiency (up to 98%), low pollution emissions (20% reduction in SO_2_ and 50% reduction in NOx), at only one-third the cost of other fuels, combining emission reduction and cost advantages. This characteristic makes it an important choice for industrial transformation in the context of increasingly strict environmental policies. China holds abundant coal reserves, and CWS technology converts coal into liquid fuel through physical processing, improving resource utilization and providing a new path for energy reserves. CWS technology is widely applied in areas such as the long-distance pipeline transportation of finely ground coal, direct combustion, and pressurized gasification. Pressurized gasification CWS is a high-concentration, finely ground slurry, typically with a concentration of 60–64% (wt) and an average particle size of less than 200 μm CWS, composed of 60–70% coal, 20–39% water, and 1% additives. It is fed into a coal mill, pressurized by a feed pump, and then introduced into four process burners. Pure oxygen from an air separation unit is divided into four streams, regulated by flow control, and fed into the process burners. The CWS and oxygen are injected into the gasifier through the process burners [2]. The accurate measurement of the CWS feed into the gasifier directly impacts its safety and economic efficiency, making the measurement of CWS flow highly significant [3].

Scholars have conducted extensive research on the performance of CWS. In 2019, Juan Xiao et al. [4] studied the effects of concentration and temperature on the flow characteristics of CWS through rheological experiments, obtaining a rheological model of coal–water slurry. The rheological model described the functional relationship between shear rate, concentration, and temperature, demonstrating good fitting properties and providing a new method for establishing empirical models of non-Newtonian fluids. In 2020, Chai et al. [5] conducted a systematic study on the vibration characteristics of CWS systems using laboratory analyzers. The results showed that as the vibration intensity increased, the stability of CWS first decreased and then increased. When the vibration intensity was further increased, the stability deteriorated again. In 2021, Harmanpreet Singh et al. [1] proposed the influence of particle size distribution on the slurry and flowability of coal–water slurry. They demonstrated how careful selection of particle size distribution can help improve the stability, rheology, and flow characteristics of the slurry. By evaluating certain parameters such as coal sample settling characteristics, the optimal coal sample particle size composition was determined to improve the flow characteristics of high ash Indian coal. In 2022, Feng et al. [6] studied the flow behavior and electrical resistivity characteristics of coal slurry under different flow rates and found that the fluctuation of electrical resistivity with time increases at higher flow rates. High resistance response zones and low resistance response zones appear at the bottom and top of the pipeline, and their range decreases with increasing flow rate and increases with increasing transport distance.

The gasification and combustion processes of CWS require the strict control of the ratio of raw materials to oxygen. For example, in the Texaco gasifier, the flow rate of CWS affects the synthesis gas (H_2_, CO) generation efficiency of the gasification reaction and participates in the safety interlock system. Flow measurement deviation may lead to oxygen imbalance, causing explosion risk or reducing gasification efficiency.

Accurate flow measurement can optimize the ratio of CWS to oxygen, reduce raw material waste, and lower production costs. For example, in a gasifier, a 1% flow error may result in economic losses of CNY tens of thousands per day. The energy security value of CWS is closely related to its industrial application, and flow measurement is the core technical link to ensure its process safety, efficiency, and economy. By adapting and optimizing the technology of flow meters, the accurate measurement of CWS can be achieved under complex working conditions, thereby supporting the implementation of national energy strategies. In the future, with the further promotion of CWS in the coal chemical industry, power generation, and other fields, intelligent and highly reliable flow measurement technology will become a key research direction.

The flow meters used for measuring the flow rate of solid–liquid two-phase flow include electromagnetic flow meters, ultrasonic flow meters, and Coriolis mass flow meters. Among these, the electromagnetic flow meter is based on Faraday’s law of electromagnetic induction and calculates the volumetric flow rate through the electromotive force generated by the cutting of magnetic induction lines by a conductive fluid. It is only suitable for conductive liquids (conductivity usually needs to be greater than 5 μs/cm) and is not suitable for gases or oils. It is not sensitive to changes in fluid temperature and density, but is susceptible to interference from bubbles or solid particles [7,8]. An ultrasonic flow meter is a type of flow meter that calculates flow velocity by measuring the time difference (time difference method) or Doppler frequency shift of ultrasound propagation in a fluid. It is suitable for clean single-phase fluids such as water and natural gas, and has exhibits accuracy but high cost [9]. The Coriolis mass flow meter is based on the Coriolis effect, which directly obtains mass flow rate and density by measuring the phase difference generated by the fluid in the vibrating tube [10]. Compared with electromagnetic and ultrasonic flow meters, the Coriolis mass flow meter can directly measure fluid mass flow rate without additional compensation (such as temperature and pressure). Coriolis mass flow meters are irreplaceable in trade settlement (such as LNG) and high-precision process control. Electromagnetic flow meters offer significant cost advantages in highly corrosive media, while ultrasonic flow meters are unique in large diameter, non-invasive scenarios. The most important difference between the three types of flow meters is that the Coriolis mass flow meter is the only instrument that can directly measure the mass flow rate of multiphase flow, as shown in Table 1.

Coriolis mass flow meters are capable of directly acquiring signals related to mass flow, offering advantages such as a wide measurement range, high accuracy, and the ability to track pulsed flows [11]. Due to their low uncertainty and ability to simultaneously measure fluid density and mass flow, Coriolis mass flow meters are widely used in industrial measurement, with single-phase measurement errors as low as 0.1%. However, significant measurement errors still occur in multiphase flow scenarios, such as in the petroleum industry [12]. Thus, applying Coriolis flow meters to multiphase flow measurement while minimizing errors has become a key challenge in this field.

According to the measurement principle of Coriolis flow meters, if the errors in multiphase flow measurement are repeatable, it is possible to develop a model to correct these errors. This theory enables the development of a multiphase flow measurement correction system based on Coriolis flow meters. In 2001, Liu et al. [13] pioneered the application of neural network algorithms to correct gas–water two-phase flow measurements. Their study, based on experimental observations of gas holdup, liquid flow rate, and gas flow rate, used a radial basis function (RBF) neural network model to correct density and mass flow measurement errors. They successfully maintained measurement errors within 2% for gas holdup, ranging from 0% to 40%, and gas–liquid two-phase flow mass flow rates ranging between 1.5 kg/s and 4.0 kg/s. This study introduced deep learning methods into the field of multiphase flow measurement using Coriolis flow meters, providing a new direction for subsequent research. In 2021, Bu Yi [14] investigated the influence of different factors on errors in oil–water–gas three-phase flow models and used BP neural networks and genetic algorithms to correct measurement errors in Coriolis mass flow meters. In 2010, Geng [15] conducted repeatability tests on Coriolis flow meters, demonstrating their excellent repeatability, which supports the use of artificial neural networks for error correction. Despite extensive research in the field of mass flow meters, most studies have focused on gas–liquid two-phase media, with limited attention to solid–liquid two-phase media.

Foreign flow meter experts have also conducted a significant amount of research in the field of flow meter fault diagnosis. For example, a turbine flow meter manufacturer has produced a mobile device for the online monitoring of turbine flow meters. This device can achieve online monitoring and temporarily collect and analyze data from the turbine flow meter to determine its operating status. However, since it is a mobile device, it is impossible to achieve uninterrupted online monitoring. In addition, this method requires operation by professional personnel, resulting in a high cost of online monitoring, with a single online monitoring cost of about CNY 20,000 per unit [16]. In addition, foreign experts have conducted in-depth research on methods for diagnosing flow meter faults. For example, Yu mei Sun et al. [17] proposed an online self-diagnosis method for flow meters, designing a flexible fault detection circuit as a state detection module on wireless sensor network nodes to diagnose flow meter faults through the relationship between instantaneous flow measurement and vibration frequency. Ning Dayong et al. [18] proposed an adaptive noise reduction method based on dislocation superposition, which can achieve the automatic noise reduction of low signal-to-noise ratio synchronous hydraulic motor signals. The adaptive noise reduction method is used to detect the health status, wear status, rust status, etc. of the flow meter. Jingqiong Zhang et al. [19] proposed using a stiffness diagnosis to identify and detect potential wear in flow meters. The driver and sensor signals were digitally processed, and the obtained frequency response was compared with the diagnostic parameters related to stiffness extracted from the flow meter, demonstrating a good method for detecting flow meter faults. Olfa Fakhfakh et al. [20] developed an indirect and incremental diagnostic method to identify the root cause in the event of a single fault. They developed a mathematical model to simulate the flow meter diagnostic method under cyclic scheduling, using constraint programming techniques to effectively diagnose flow meter faults. Ruixiang Deng et al. [21] proposed a novel fault diagnosis algorithm based on fault-related variable selection. From the normal state to the fault state, the least absolute shrinkage and selection operator (LASSO) was used to analyze the relative changes between variables and statistics. In order to determine the optimal set of fault-related variables, a fault reconstruction algorithm, based on least angle regression (LAR), was proposed. Chen et al. [22] proposed a method for isolating flow meter faults in centralized cooling systems for buildings, mathematically explaining the existence of collinearity in flow meter faults in centralized cooling systems. Wavelet transform was used as an alternative method to isolate flow meter faults. Zhou et al. [23] proposed a smart energy meter fault diagnosis model (DBN-CapsNet) based on an improved capsule network (CapsNet) for the historical fault data information of smart energy meters. The impact of batch size, optimizer, and iteration times of the capsule network on the fault diagnosis performance of smart energy meters was discussed. L. Ren et al. [24] proposed a single-sensor early fault detection method based on wavelet transform and kernel principal component analysis (KPCA), demonstrating the accuracy and effectiveness of this method in detecting early faults in single sensors through experiments. Liuen Guan et al. [25] established an incremental fault diagnosis (IFD) framework and further proposed a model evolution mechanism based on adaptive knowledge distillation (KD) and representative sample selection (MEMAR). Experimental results showed that the proposed MEMAR can reduce the storage space and training time of old samples. In addition, it enables deep learning-based fault diagnosis models to display excellent diagnostic performance for both new and old samples.

Several reports [26,27] have discussed theoretical studies on errors in Coriolis tubes for different types of mixtures, including slurries. While these studies provide a theoretical understanding of errors, practical implementation in slurry flow measurement remains lacking. With the rapid development of artificial intelligence and machine learning algorithms, data-driven models have been increasingly applied in various fields. In recent years, deep learning, a branch of artificial intelligence, has achieved revolutionary progress in fields such as image recognition, natural language processing, and pattern recognition. Its powerful data processing capabilities and ability to learn complex patterns from large datasets offer new solutions for flow measurement.

This study aims at utilizing deep learning to address the measurement inaccuracies encountered when using Coriolis mass flow meters for measuring the flow rate of coal–water slurry, a liquid–solid two-phase flow. By constructing and training neural network models, we seek to achieve the accurate measurement of fluid flows of varying types and complexities. We will begin by outlining the fundamental principles of Coriolis mass flow meters, followed by the establishment of an experimental platform for coal–water slurry liquid–solid two-phase flow. Finally, we will detail the design, training, and validation processes of the deep learning model.

This research provides a new technical approach for Coriolis mass flow meters in the field of liquid–solid two-phase flow measurement, aiming at enhancing measurement accuracy, reducing costs, and offering more reliable data support.

## 2. Measurement Principle for Dual-Tube U-Shaped Coriolis Mass Flow Meter

Coriolis flow meters can be classified into various types based on different criteria. According to the shape of the measuring tube, they can be divided into those with bent tubes and those with straight tubes, with bent tubes further categorized into U-shaped tubes, Ω-shaped tubes, and ∆-shaped tubes. Based on the number of measuring tubes, they can be classified as single-tube or dual-tube types. Depending on the material of the measuring tube, they can be made of Hastelloy C-22 alloy, 316L stainless steel, titanium, 904L stainless steel, tantalum, and others. In this experiment, a dual-tube U-shaped Coriolis mass flow meter made of 316L stainless steel was selected. The dual-tube structure was chosen because when both tubes operate at the same resonant frequency, it not only effectively reduces external interference but also enhances continuous resonance in the measurement area, providing excellent signal gain. Additionally, the U-shaped tube design facilitates easier vibration initiation and reduces the requirements for material stiffness. Furthermore, the dual-tube design requires a lower driving current.

The dual-tube U-shaped Coriolis mass flow meter consists of two main components: the flow sensor (primary device, also known as the primary instrument) and the flow converter (auxiliary device, also known as the secondary instrument). The flow sensor is composed of two measuring tubes, a driver, two sensors, and supporting components. When no fluid flows through the tubes, the oscillation frequency generated by the driver installed in the middle of the tubes matches the natural frequency of the tubes, resulting in no phase difference. However, when fluid flows through the measuring tubes, the Coriolis effect causes the tubes to vibrate, producing a phase difference detectable by the sensors at both ends of the tubes. This phase difference is proportional to the mass flow rate of the fluid. By measuring the phase difference and signal frequency, the mass flow rate can be determined. The flow converter is responsible for converting the signals detected by the sensors into mass flow rate and providing the necessary drive for the sensors.

In a rotating reference frame, when an object undergoes translational motion relative to the frame, it experiences not only a centripetal force but also an additional force known as the Coriolis force. By utilizing a precision rotating disk and the flow characteristics within it, accurate flow measurement can be achieved. On this disk, the weight of a particle is referred to as the inertial force, whose magnitude determines the trajectory of the object. Therefore, the motion state of the object can be reflected by measuring the magnitude of the inertial force.

(1) $$F_{C}=2mv\omega$$

where *F_C_* represents the Coriolis force, *m* represents the mass of the measured particle, *ω* represents the angular velocity, and *v* represents the radial velocity in the rotating or oscillating system.

**Figure 1:**
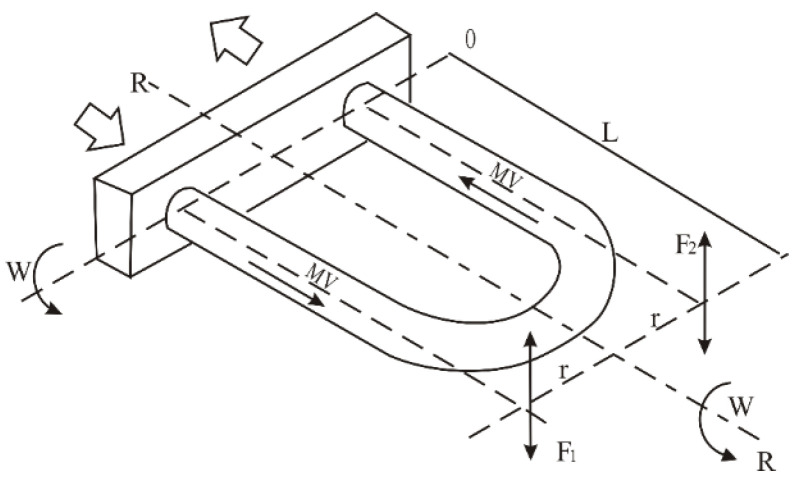
Sensor measurement tube motion and force diagram.

**Figure 2:**
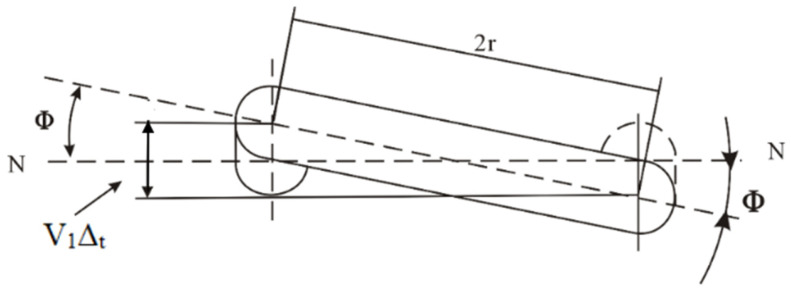
Schematic diagram of Coriolis force causing twisting deformation of U-shaped tube.

The Coriolis force possesses significant strength, and its direction of action is entirely consistent with the measured object. Utilizing the power of an electronic driver, the presence of the Coriolis force causes a change in the trajectory of the fluid, generating a relative force, as illustrated in Figure 1. Due to the differing pressures experienced at the two ends of the U-shaped tube, it is subjected to a torque. When the U-shaped tube is influenced by vertical vibrations, it exhibits noticeable alternating torsional deformation, as shown in Figure 2.

Among them, *R* is the axis of symmetry, *ω* is the angular velocity, *F*_1_ and *F*_2_ are the directions of Coriolis force, *θ* is the torsion angle, and ∆*t* is the time difference.

The torque *M* is directly proportional to the Coriolis force, and Coriolis force is in turn directly proportional to mv. Therefore, by measuring the twist angle of the U-tube and calculating the torque *M*, one can determine the mass flow m. Since the precise measurement of the twist angle is challenging to achieve, utilizing the relationship between the twist angle and the time difference ∆*t*, the time required for the parallel straight tubes at both ends of the U-tube to pass through the center equilibrium point can indirectly measure the angle *θ*, thereby realizing the measurement of mass flow.

Assuming that the unit mass of the fluid under test is m and the time taken to flow through a unit length is t, the mass flow rate can be expressed as $Q_{m}=m/t$. Given the fluid velocity as v=L/t, the torque T acting on the sensing tube during deformation is as follows:

(2) $$T=2F_{c}r=4m\omega vr$$

Considering the relationship between mass flow rate and fluid velocity, the velocity can be rewritten as $v=LQ_{m}/m$. Simultaneously, let the elastic coefficient of the sensing tube be K, such that the sensing tube in the operational state satisfies T=K·θ. Substituting these into the above equation yields the following:

(3) $$Q_{m}=\frac{K\theta }{4\omega rL}$$

## 3. Design of the Coal–Water Slurry Solid–Liquid Two-Phase Flow Measurement Platform

Based on the weighing method, this paper designs a measurement platform suitable for the flow rate measurement of coal–water slurry (CWS) solid–liquid two-phase flow. This platform can provide standards for both single-phase water and solid–liquid two-phase CWS. The hardware of the solid–liquid two-phase flow platform consists of instruments and equipment, control cabinets, and screw pumps. The main instruments and equipment include pressure transmitters, flow meters, electronic scales, etc.

The weighing method offers high measurement accuracy and can directly calibrate the mass flow rate of fluids. The solid–liquid two-phase flow measurement platform is designed with a weighing reference system, which mainly consists of a three-way valve, a weighing container, an electronic scale, hoses, and stainless steel rigid pipes. Among these, the measurement accuracy of the electronic scale is the primary factor affecting the calibration precision of the weighing method, and the weighing system of the solid–liquid two-phase flow platform uses a high-precision electronic scale.

**Figure 3:**
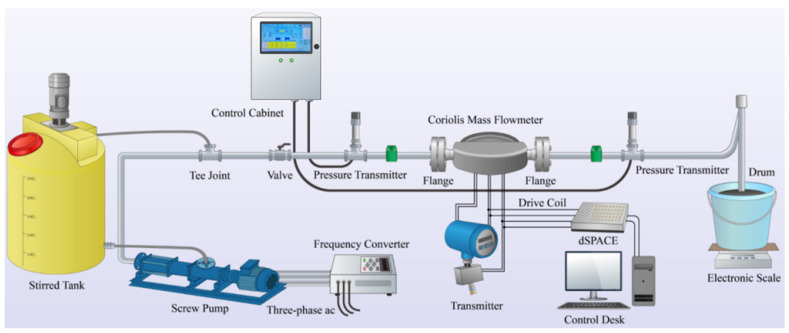
Experimental platform for coal–water slurry based on Coriolis mass flow meters.

The field experimental setup for this project, which is composed of a dSPACE hardware system, ControlDesk software, two dual U-tube Coriolis mass flow meters with inner diameters of 8 mm (from a certain company), and water supply equipment, is shown in Figure 3.

**Figure 4:**
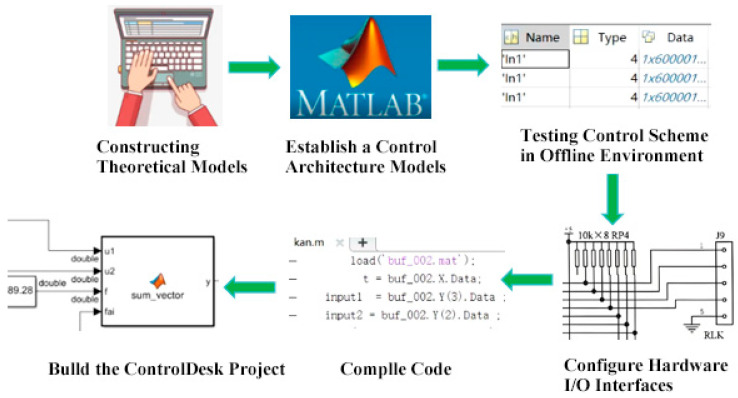
DSPACE control scheme diagram.

The dSPACE real-time hardware-in-the-loop (HIL) simulation system used in this paper is a development platform that seamlessly integrates with MATLAB/Simulink, designed for the development and validation of control algorithms for drive systems, its control scheme is shown in Figure 4. The dSPACE software system primarily consists of three components: ControlDesk (host software), RTI (Real-Time Interface) Library, and MATLAB/Simulink (digital simulation software). The MATLAB/Simulink is used to build the control systems and perform offline simulations. The RTI Real-Time Interface Library configures dSPACE hardware interfaces to enable the real-time processing of feedback pulse signals. ControlDesk, the host software, loads the SDF file generated by Simulink compilation and provides real-time monitoring and control during the experiments. The development workflow for dSPACE control algorithms is outlined below, with the following key steps:

Using the MATLAB/Simulink software platform, a data acquisition architecture is constructed, and numerical simulations are completed in an offline environment.

The Z15 series mass flow sensor was configured with the RTI (Real-Time Interface) Library’s I/O module, operating at a sampling rate of 10 kHz with a sampling interval of 100 μs, to establish communication between dSPACE’s software and hardware. Subsequently, the data acquisition program was compiled using the MATLAB/Simulink software platform, and the generated SDF file was loaded into the dSPACE system.

The virtual monitoring interface was constructed in the ControlDesk host software to enable the parameter adjustment and real-time monitoring of the control system.

### 3.1. Coriolis Flowmeter Water Calibration Experiment

To test the accuracy of the Coriolis mass flow meter proposed in this paper for the transportation of coal–water slurry, an on-site experimental test was conducted using the Dongfeng Electromechanical Z15 type Coriolis mass flow meter. The measuring tube material is made of 316L stainless steel, with a wall thickness of 2.5 mm, and the outer diameter of each tube in the dual-tube system is 10.37 mm.

To verify the accuracy and reliability of the phase difference prediction of the theoretical model, it is first necessary to use a water calibration experiment to verify the accuracy and stability of the flow detection provided by the DPT100 type transmitter that accompanies the Coriolis mass flow meter. Only then can the mass flow rate and phase difference information recorded in the transmitter be read.

The water calibration experimental testing platform for the Coriolis mass flow meter built for this experiment is shown in Figure 3. The experimental platform mainly includes a frequency converter water pump motor, a pneumatic valve, a temperature sensor, a calibrated meter, a pressure sensor, and a control valve; water is used as the measuring liquid, with a density of 998 kg/m^3^, and the size of the flow is controlled through a real-time liquid flow monitoring system.

During the calibration process, the deviation of each actual calibration flow rate from the set flow rate at each flow point must not exceed ±5% of the set flow rate. The calibration is conducted three times at each flow point. The calibration results are compared, as shown in Table 2.

From the analysis of the calibration results, it can be seen that the maximum measurement error of this Coriolis mass flow meter is 2.89‰, and the measurement accuracy of each test is within three thousandths. The maximum repeatability is only 0.95‰, which proves that the use of the accompanying transmitter displays a high measurement accuracy and stability when working on this calibration platform, meeting the experimental verification requirements.

Corresponding specific experimental steps: Firstly, install the Coriolis mass flow meter onto the water pipeline to ensure that the entire device is connected correctly and can work normally. Connect the Coriolis mass flow meter to the DSPACE real-time control work platform, open the experimental device, adjust the flow control valve to the maximum, and pass CWS through the device for 3 min to ensure that the fluid in the sensitive pipe is coal–water slurry; then, establish a Simulink drive control algorithm diagram, set the sampling frequency to 10 kHz, and determine the sampling time based on the experimental state. Start the DSPACE real-time control platform, complete the drive control methods mentioned in the experimental plan, and save the experimental data. In order to verify the influence of the flow rate on the vibration signals, the same driving control method was selected, and the flow rate was adjusted through the flow control valve to complete the signal acquisition experiment for flow rate under different densities, temperatures, and flow rates. The weighing values and flow rate values displayed on the meter were read separately. Finally, close the DSPACE real-time control work platform and flow control valve separately to end the experiment.

### 3.2. Experimental Process and Results

This experiment employs the weighing method. Since the density of coal–water slurry (CWS) in actual production is approximately between 1.1–1.26 kg/m^3^, multiple samples of CWS were collected from a plant in Shenmu, Yulin, Northern Shaanxi, for this experiment. Four different densities of CWS were selected for the experiment, i.e., 1.196 kg/m^3^, 1.215 kg/m^3^, 1.181 kg/m^3^, and 1.153 kg/m^3^. Under each different density of CWS, various temperatures were altered, followed by changes in flow velocity. A total of 100 sets of experiments were conducted, and then the experimental data were organized, resulting in the integration of 90 sets of data. Subsequently, the Coriolis mass flow meter was driven and controlled using the DSPACE real-time control platform and ControlDesk software. The collected experimental data were imported into MATLAB for numerical analysis to derive the phase difference.

In the experiments involving the liquid–solid two-phase flow of coal–water slurry, measurements included pressure difference, flow velocity, temperature, density, phase difference, and time. These measured factors can effectively reflect the influencing factors during the experimental process. The measurement results are shown in Table 3.

### 3.3. Error Analysis

The errors of the Coriolis mass flow meter mainly originate from the following aspects:

1. Phase Difference

The phase difference of the Coriolis mass flow meter is one of the primary indicators affecting the performance parameters of the instrument. The larger the phase difference, the more accurate the measurement results [28]. There are many factors that affect the phase difference, such as the position of the detection points on the measuring tube in the primary instrument and the additional mass of the exciter and signal detector. In the secondary instrument, the selection of the sensor, the design of the hardware system, and the study of the signal phase difference detection algorithm are additional influencing factors. The shape parameters of the detection tube are also important factors affecting the phase difference. Additionally, the signal phase difference (i.e., time difference) information affects the accuracy of the Coriolis mass flow meter because there are often errors in the secondary instrument’s detection of the signal phase difference. These include errors caused by the detection accuracy of the system algorithm, known as systematic errors, as well as random errors inherent in the secondary instrument’s detection; however, an increase in the phase difference between the two signal paths can reduce the impact of the total detection error caused by systematic and random errors. Therefore, the larger the phase difference, the more conducive it is to improving the instrument’s interference resistance and detection accuracy [29].

2. Pressure Duration

During the operation of the Coriolis mass flow meter sensor, significant changes in the medium’s pressure can affect the measurement accuracy [30]. The impact of pressure varies for different types of Coriolis mass flow meters. The mass flow verification equation for the U-tube is given by Equation (4) [31].

(4) $$q_{m}=\frac{k_{s}}{8r^{2}}\times ∆t$$

where *q_m_* represents the mass flow rate; *k_s_* represents the torsional stiffness of the sensor’s U-shaped measuring tube; *r* represents the radius of the measuring tube; represents the time interval for the detection points to pass through the center of vibration. An increase in working pressure causes the vibrating measuring tube to be under tension, and there is also a Bordon tube effect, where an increase in the value of r (the radius of the measuring tube) will cause the measured value to decrease.

3. Temperature

In 2003, Patten [32] described an operating method for flow meters at low temperatures, which essentially involves a linear correction for temperature-dependent changes in Young’s modulus. In 2021, Asaad, K. et al. [33] reported the results of measuring LNG with a Coriolis mass flow meter, noting that when a linear compensation for Young’s modulus was applied at low temperatures, the measurement error was very close to that when measurements were taken at ambient temperatures. In 2009, Tao, W. et al. [34] concluded through experiments that the nonlinear Young’s modulus and thermal expansion of the measuring tube material can affect measurement accuracy. In 2012, Miao, L. et al. [35] pointed out in their co-authored article that changes in fluid temperature can affect the changes in the elastic modulus of the test tube, thereby affecting the meter coefficient and measurement accuracy of the flow meter.

The impact of temperature effects on the accuracy of the Coriolis flow meter is reflected in the nonlinear changes in the elastic modulus and Poisson’s ratio of the measuring tube material caused by temperature variations. The main source of error in the measurement of the Coriolis mass flow meter comes from the axial force in the measuring tube, and one of the main reasons for the generation of the axial force is the influence of temperature. The relationship between the axial force and temperature is given by Equation (5).

(5) $$F_{x}=\pi \left(b_{0}^{2}-a_{0}^{2}\right)E\alpha ∆t$$

where ∆*t* is the change in temperature; *a*_0_ is the inner diameter of the test tube; *b*_0_ is the outer diameter of the test tube; *E* is the elastic modulus of the test tube; α is the coefficient of thermal expansion of the material of the test tube.

Additionally, temperature directly affects the frequency and measurement accuracy, primarily through causing changes in the elastic modulus. The elastic modulus *E* is a function of temperature.

(6) $$E=E(t)=E_{0}\left[1-\alpha \left(t-t_{0}\right)\right]$$

4. Flow Velocity

The natural frequency of the fluid transport pipeline decreases as the fluid velocity increases. When the flow velocity reaches the critical velocity of the pipeline, the pipeline will lose its stability. Tong, M. et al. [36] used the QR method to analyze the relationship between the pipeline resonance frequency and fluid flow velocity. The analysis results show that changes in fluid flow velocity can cause changes in the pipeline resonance frequency.

Different flow velocities of the fluid inside the measuring tube of the Coriolis mass flow meter can lead to changes in the resonance frequency of the measuring tube, thereby causing inaccuracies in the density measurement of the Coriolis mass flow meter [37]. Based on the above theory, Equation (7) can be derived.

(7) $$q_{m}=vA_{l}\rho_{l}$$

From Equation (7), the following can be deduced:

(8) $$v=\frac{q_{m}}{A_{l}\rho_{l}}$$

where *v* represents the fluid velocity; *A_l_* represents the cross-sectional area of the fluid; *ρ_l_* represents the density of the fluid.

5. The Relationship Between Temperature, Pressure Difference, and Mass Flow Rate

The mass flow rate of a fluid is related to temperature and pressure. Using the theory of “multiple nonlinear regression”, assuming the experimental indicators are mass flow rate y, pressure difference x_1_, and temperature x_2_, then they approximately satisfy the quadratic regression model, as follows:

(9) $$\overset{\wedge }{y}=a+\sum_{j=1}^{2}b_{j}x_{j}+\sum_{j=1}^{2}b_{jj}x_{j}^{2}+\sum_{j<k}b_{jk}x_{j}x_{k}=a+b_{1}x_{1}+b_{2}x_{2}+b_{11}x_{1}^{2}+b_{22}x_{2}^{2}+b_{12}x_{1}x_{2}$$

Assume: X_1_ = x_1_, X_2_ = x_1_^2^, X_3_ = x_1_x_2_, X_4_ = x_2_, X_5_ = x_2_^2^;

B_1_ = b_1_, B_2_ = b_2_, B_3_ = b_11_, B_4_ = b_22_, B_5_ = b_12_.

After organizing and calculating the raw experimental data, the normal equation system can be obtained as follows:

(10) $$\left\{\begin{array}{c} \bar{y}-B_{1}\bar{X_{1}}-B_{2}\bar{X_{2}}-B_{3}\bar{X_{3}}-B_{4}\bar{X_{4}}-B_{5}\bar{X_{5}}=a\\ L_{11}B_{1}+L_{12}B_{2}+L_{13}B_{3}+L_{14}B_{4}+L_{15}B_{5}=L_{1y}\\ L_{21}B_{1}+L_{22}B_{2}+L_{23}B_{3}+L_{24}B_{4}+L_{25}B_{5}=L_{2y}\\ L_{31}B_{1}+L_{32}B_{2}+L_{33}B_{3}+L_{34}B_{4}+L_{35}B_{5}=L_{3y}\\ L_{41}B_{1}+L_{42}B_{2}+L_{43}B_{3}+L_{44}B_{4}+L_{45}B_{5}=L_{4y}\\ L_{51}B_{1}+L_{52}B_{2}+L_{53}B_{3}+L_{54}B_{4}+L_{55}B_{5}=L_{5y} \end{array}\right.$$

Get:

$$\left\{\begin{array}{c} 2317-0.565B_{1}-0.35B_{2}-25.6B_{3}-45.43B_{4}-2217.6B_{5}=a\\ 2561B_{1}+2.26B_{2}+123.25B_{3}+2.22B_{4}+31.32B_{5}=153.96\\ 2.2625B_{1}+971.895B_{2}+111.46B_{3}+6B_{4}+137.39B_{5}=262.46\\ 2123.25B_{1}+111.46B_{2}+10,734.6B_{3}+8125.56B_{4}+685,572B_{5}=260,738.88\\ 2.22B_{1}+6B_{2}+8125.56B_{3}+13,835.9B_{4}+1,160,104.64B_{5}=13,835.9\\ 31.32B_{1}+137.39B_{2}+685,572B_{3}+1,160,104.64B_{4}+98,869,891.56B_{5}=32,813,667.2 \end{array}\right.$$

Get the solution:

a = −6245.5 B_1_ = −604.5 B_2_ = −1414.74 B_3_ = 12,704.73

B_4_ = −6245.5 B_5_ = −14.48

So the sixth variable linear regression equation is obtained as follows:

y = −604.5X_1_ − 1414.74X_4_ + 12,704.73X_2_ − 6245.5X_5_ − 14.48X_3_ − 6245.5

Through the significance test of the linear regression equation, it can be seen that the established linear regression equation is very significant and fits well with the experimental data.

Therefore, due to the approximate functional relationship between the experimental indicator quality flow rate y and the pressure difference x_1_, the temperature x_2_ is as follows:

y = −604.5x_1_ − 1414.74x_2_ + 12,704.73x_1_^2^ − 6245.5x_2_^2^ − 14.48x1x_2_ − 6245.5 (11)

## 4. Error Correction Based on BP Artificial Neural Network

In recent years, various advanced algorithms have been explored for error correction in flow measurement. Radial basis function (RBF) neural networks have been applied in correcting gas–water two-phase flow measurements. RBF networks [38], known for their fast learning speed and good generalization ability, can effectively approximate nonlinear functions. They have shown promising results in flow measurement error correction with their unique advantages in handling complex nonlinear relationships. On the other hand, support vector machines (SVMs) [39], based on statistical learning theory, aim to minimize the generalization error bound by solving convex optimization problems. They have been successfully applied in many fields, including flow measurement. SVMs can effectively avoid overfitting and provide good prediction accuracy, even with limited training data, by mapping the input data into a high-dimensional feature space and constructing an optimal separating hyperplane.

These methods, including RBF networks and SVMs, have contributed significantly to the advancement of flow measurement technology. However, in this study, we chose to focus on the BP neural network as the primary correction method. The BP neural network, with its strong nonlinear mapping capability and adaptability, has been widely used in many complex systems. In our preliminary experiments and analyses, the BP neural network demonstrated good potential for correcting the measurement errors of coal–water slurry liquid–solid two-phase flow. Furthermore, the combination of a BP neural network with other algorithms like XGBoost in our optimized model has led to a significant improvement in prediction accuracy.

The measurement error of coal–water slurry solid–liquid two-phase flow is related to a variety of variables and is complex and nonlinear, making it difficult to study from a mechanistic analysis perspective. Based on test data, this paper establishes an empirical model to find a nonparametric framework to represent the implicit functional relationship between inputs and outputs.

On the basis of regularity analysis, a correction method for multiphase flow measurement results based on deep learning will be introduced, which will control the corrected error within a certain range. The dataset used in this experiment comes from the coal–water slurry solid–liquid two-phase flow experiment, including a total of 90 sets of measured data. Each set of data includes pressure difference (MPa), flow velocity (m/s), fluid temperature (°C), density (kg/m^3^), phase difference (s), time (s), and display (g), and other element information.

In this experiment, the training platform used is a Core i5 8th Gen CPU and an NVIDIA GTX 1050ti GPU. In the ResNet part, we use 256 filters, a convolution kernel size of 2, and a stride of 1. In the LSTM part, we set up two LSTM layers, with the first layer using 2000 LSTM units and the second layer using 1000 LSTM units, with the activation function for each layer being the ReLU function. During the LSTM training phase, we set the number of iterations to 100 and the batch size to 16; the optimizer used during the training process is the Adam optimizer, with the loss function being the mean squared error (MSE) loss function.

### 4.1. Initial BP (Backpropagation)

In this paper, we use CNN and LSTM as our network’s backbone. The selection of CNN and LSTM in this study is based on their unique advantages in handling different types of data and tasks. CNN, with its ability to automatically learn hierarchical features from raw data through convolutional layers, is particularly effective in capturing local features and spatial correlations in the input data. This makes it suitable for extracting meaningful features from the complex and nonlinear relationships present in the coal–water slurry flow measurement data. On the other hand, LSTM is a specialized RNN architecture designed to address the vanishing gradient problem and capture long-term dependencies in sequential data. Since the flow measurement data used in this study can be considered as sequential data with temporal correlations, LSTM is capable of extracting both short-term and long-term dependency features, which helps improve the model’s ability to accurately generalize and predict.

**Figure 5:**
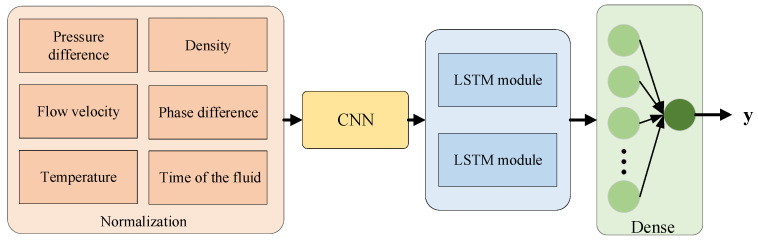
Initial BP network architecture.

The network model designed for predicting the weighing readings of coal–water slurry is shown in Figure 5. First, the input data $X=[x_{1},x_{2}\cdots x_{6}]$ (where $x_{1},x_{2}\cdots x_{6}$ represent the pressure difference, flow velocity, temperature, density, phase difference, and time of the fluid, respectively); after normalization [40], the data is processed to obtain $X^{0}=\left[x_{1}^{0},x_{2}^{0}\ldots x_{6}^{0}\right]$. Normalization processing makes the data distribution more concentrated, facilitating subsequent calculations. Then, the normalized input data are passed through a convolutional module [41], two LSTM modules [42], and a fully connected layer [43] to deeply extract the data features and thereby achieve regression prediction of the weighing data. Next, we will introduce the specific structure of each module in detail.

#### 4.1.1. Convolutional Module (CNN)

**Figure 6:**
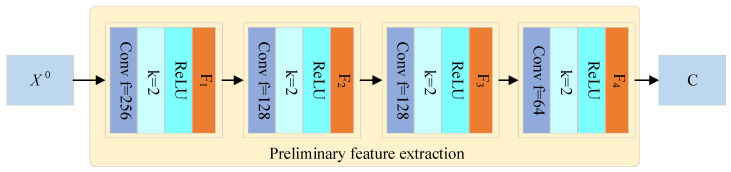
Structure of the CNN module.

A convolutional neural network (CNN) is a type of deep learning architecture widely used in image and video recognition, classification, and segmentation tasks. By simulating the working principle of the human visual system, CNNs can automatically learn features from input data. As a feedforward neural network, CNNs use convolutional layers to automatically and hierarchically extract features. Unlike traditional machine learning methods, CNNs do not require manual feature engineering because they can learn effective feature representations from raw data [44]. In this paper, to capture the relationship between the aforementioned six factors and the weighing results and to improve the network’s feature extraction capability and complexity, we first input the normalized data into four cascaded convolutional layers for preliminary feature extraction, as shown in Figure 6. The first convolutional layer includes 512 convolution kernels, the second layer includes 256 convolution kernels, the third layer includes 128 convolution kernels, the fourth layer includes 64 convolution kernels, and the fifth layer includes 32 convolution kernels, with each convolution kernel size being 2 and the activation function being the ReLU function [45]. After passing through the CNN module, the initial features are obtained as follows:

(12) $$C=\sigma \left(WX^{0}+b\right)$$

where σ represents the ReLU (rectified linear unit) activation function, W denotes the weight parameters within the CNN layers, and b signifies the bias term.

#### 4.1.2. Long Short-Term Memory Module (LSTM)

**Figure 7:**
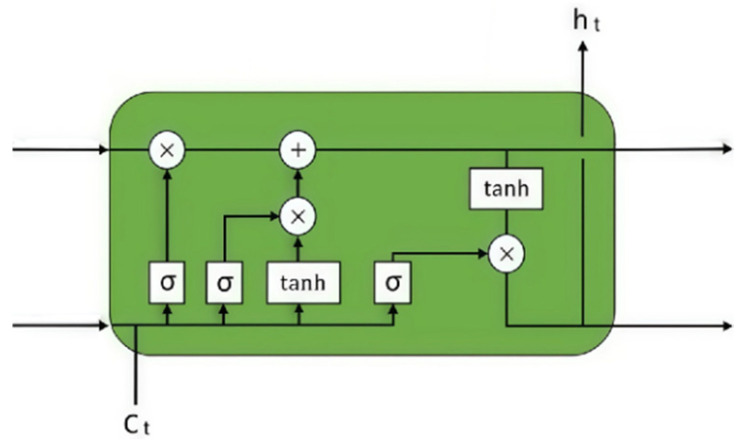
Long short-term memory module.

LSTM (long short-term memory) is a special type of recurrent neural network (RNN) architecture that was proposed by Sepp Hochreiter and Jürgen Schmidhuber in 1997 [42]. LSTM is designed to address the issues of vanishing or exploding gradients that traditional RNNs [46] encounter when dealing with long sequence data [47], especially in learning long-term dependencies. Through gating mechanisms, particularly the forget gate, LSTM has the ability to maintain long-term dependencies, allowing the network to learn dependencies across long sequences and solve the vanishing gradient problem in traditional RNNs. Because LSTM can learn long-term dependencies, it usually generalizes better to unseen data rather than merely memorizes the training data. Additionally, the forget gate and input gate of LSTM can learn when to ignore or update information, and the output gate controls the output of information. This flexibility makes LSTM adaptable to various data characteristics. The LSTM module is shown in Figure 7. Since the input data in this experiment is six-dimensional physical data, it can be considered as sequential data. Due to LSTM’s good feature extraction capability for sequential data, we introduce LSTM layers to improve the network’s feature extraction and generalization capabilities. Here, we sequentially introduce two LSTM layers (the first LSTM layer consists of 2000 LSTM units, and the second LSTM layer consists of 1000 LSTM units, with both LSTM layers using the ReLU activation function) to cascade and extract long- and short-term dependency features, as follows:

(13) $$h_{t}^{1}=\sigma \left(W_{h_{1}}\cdot h_{t-1}^{1}+W_{r1}C+b_{h_{1}}\right)$$

(14) $$h_{t}^{2}=\sigma (W_{h_{2}}\cdot h_{t-1}^{2}+W_{r2}h_{t}^{1}+b_{h2})$$

where $h_{t}^{i}(i=1,2)$ represents the hidden state of the i-th LSTM layer at time step t (here, it means the output features of the i-th LSTM layer), $W_{h_{i}}(i=1,2)$ is the weight matrix of the i-th LSTM layer used for the hidden state $h_{t-1}^{i}$ of the previous time step, $W_{r_{i}}(i=1,2)$ is the weight matrix of the i-th LSTM layer used for the input data at the current time step, $b_{h_{i}}(i=1,2)$ is the bias term of the hidden state of the i-th LSTM layer, and σ( ) is the ReLU activation function. For convenience, we will assume that the long- and short-term dependency features obtained after passing through two LSTM layers are as follows:

(15) $$H=h_{t}^{2}$$

After obtaining the long- and short-term dependency features, we need to use a fully connected layer to perform regression on the learning target (fluid weighing), as follows:

(16) $$y=D_{\mathrm{ense}}(H)$$

where y represents the result of the regression, that is, the preliminary prediction of the weighing. $D_{ense}(H)$ represents the fully connected operation.

**Figure 8:**
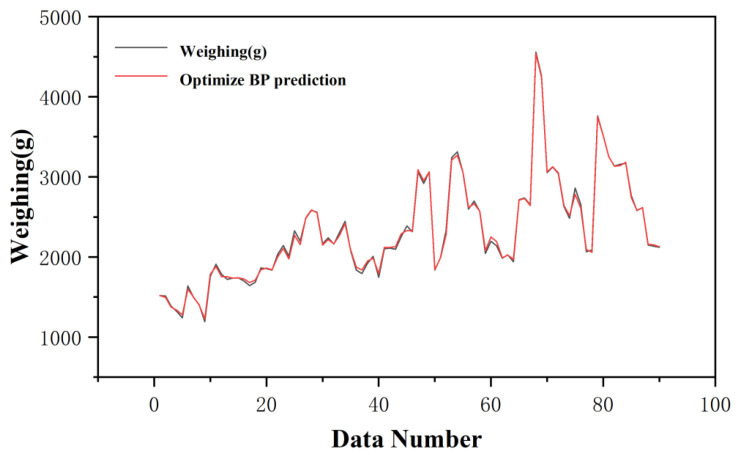
Original BP prediction results.

The prediction results are shown in Figure 8, where red represents the actual weighing results, and blue indicates the predicted results of the model designed in this paper. By calculating the metric (Equation (12)), the correction error of this prediction model is calculated to be 3.98%, which is a significant reduction compared to the error value of 5.11% measured by the DPT100 type transmitter. Although the prediction results are already better than the calculated results, there is still some difference from the actual weighing value. Therefore, further optimization of this model was carried out, as shown in Section 2.

(17) $$\frac{\left|D_{\mathrm{Weighing}\;\mathrm{value}}-F_{\mathrm{Predictive}\;\mathrm{value}}\right|}{D_{\mathrm{Weighing}\;\mathrm{value}}}\langle \frac{\left|D_{\mathrm{Weighing}\;\mathrm{value}}-E_{\mathrm{Displayed}\;\mathrm{value}}\right|}{D_{\mathrm{Weighing}\;\mathrm{value}}}$$

### 4.2. Optimized BP

**Figure 9:**
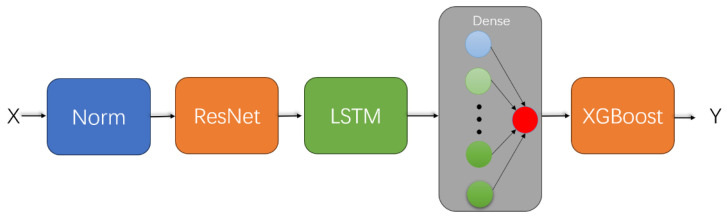
Optimized BP network architecture.

Based on the initial BP model, in order to further utilize the raw input features and improve the model’s predictive accuracy and generalization ability, we replaced the original CNN module with a ResNet module and added a tree-based model, XGBoost, to the backend of the initial BP model. By combining the feature extraction capabilities of the deep learning model (BP) with the strong regression capabilities of the tree model, we further enhanced the robustness and generalization ability of the original model. The optimized BP model is shown in Figure 9, and the specific details of the improvements are described in Section 4.2.1 and Section 4.2.2

#### 4.2.1. Residual Module (ResNet)

**Figure 10:**
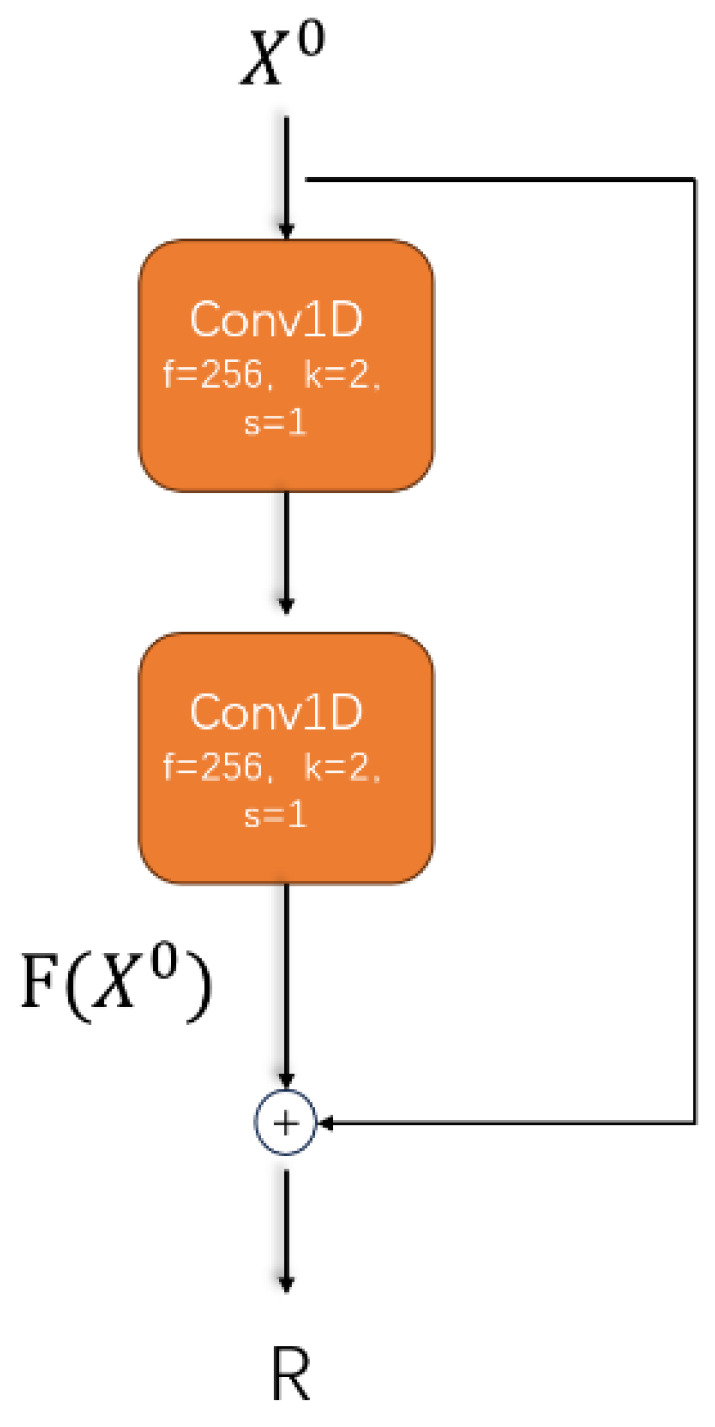
Residual module.

Residual networks [48] (ResNet), proposed by Kaiming He and others at Microsoft Research in 2015, is a type of deep learning architecture. ResNet is particularly suitable for situations that require training very deep network structures, addressing the issues of training difficulties and performance degradation as the network depth increases. As shown in Figure 10, in the model designed in this paper, we replaced the original CNN module with a residual module, allowing the network to train deeper models, thereby improving the network’s performance and generalization ability, alleviating the vanishing and exploding gradient problems, and enhancing the network’s expressive power. The residual structure allows the output of each layer to depend not only on the transformation of the current layer but also to retain the feature information of the input. This design enhances the network’s expressive ability, enabling it to better capture and learn complex features. After passing through the residual module, we obtain the hierarchical features, as follows:

(18) $$R=F(X^{0})+X^{0}$$

where $F(X^{0})$ represents the result of applying two one-dimensional convolutions. In this paper, both convolutions are set with a kernel size of 2 and a stride size of 1, and the number of kernels is set to 256.

XGBoost (extreme gradient boosting) is an ensemble learning algorithm based on gradient boosting decision trees. It is an optimized distributed gradient boosting library that uses tree algorithms to solve various machine learning problems, including classification, regression, and ranking problems, and is a member of the gradient boosting [49,50] family. Developed by Tianqi Chen and others around 2014, XGBoost quickly became a popular tool in machine learning competitions and industrial applications. XGBoost is based on the gradient boosting decision tree (GBDT) algorithm, which iteratively adds new weak prediction models (usually, decision trees) to minimize a differentiable loss function. It incorporates L1 and L2 regularization terms in the loss function, which helps control model complexity and reduce overfitting. Additionally, XGBoost further prevents overfitting by pruning trees during growth. In this paper, we construct a model with greater robustness and generalization ability by combining the feature extraction capabilities of deep learning models with the powerful regression capabilities of tree models. The combination of different models can reduce the risk of overfitting and improve performance across different data distributions. Moreover, tree models excel at handling non-linear relationships. Therefore, by introducing the XGBoost tree model to further process the high-dimensional features extracted by the model, we can better capture complex non-linear relationships in the data, thereby improving prediction accuracy.

**Figure 11:**
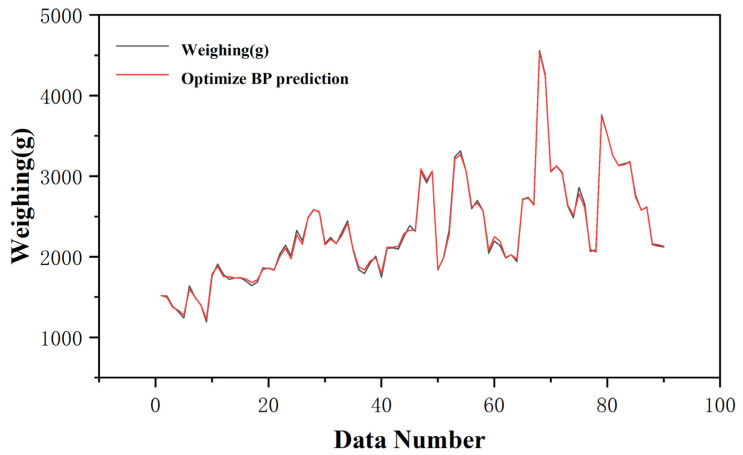
Optimized BP prediction results.

**Figure 12:**
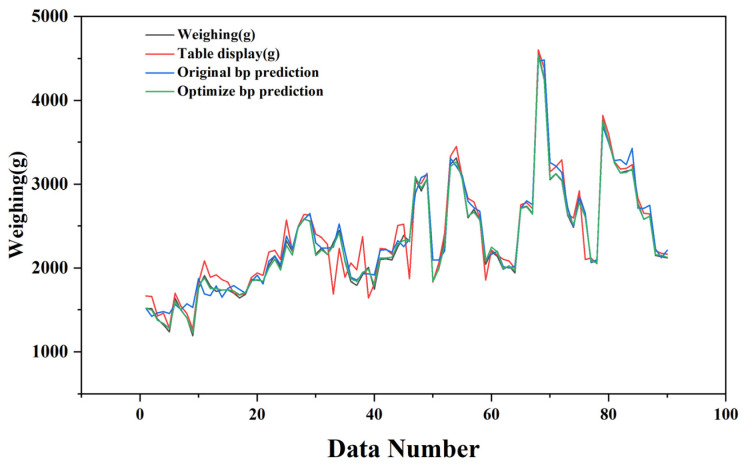
Comparison chart of actual weighing value, displayed weighing value, initial BP prediction value, and optimized BP prediction value.

This process can be represented as follows:

(19) Y=XGB(y)

where Y represents the final regression result, that is, the final prediction of the weighing, and XGB( ) denotes the regression operation performed using the XGBoost model to obtain the final regression outcome. With this, we have achieved the final improved regression result Y. The final prediction result curve is shown in Figure 11, where the corrected error of the optimized prediction model is controlled within 1.01%. It can be observed that the prediction result curve of the optimized BP model has been fitted to the actual weighing values, significantly enhancing the prediction accuracy. The final results are reflected in Figure 12.

#### 4.2.2. Experiment

In the experiments, the dataset used consists of 90 sets of coal–water slurry solid–liquid two-phase flow measurement data. To ensure the reliability and generalization ability of the model, the dataset was split into training and validation sets in a ratio of 8:2. Cross-validation was performed to further verify the stability and performance of the model.

**Figure 13:**
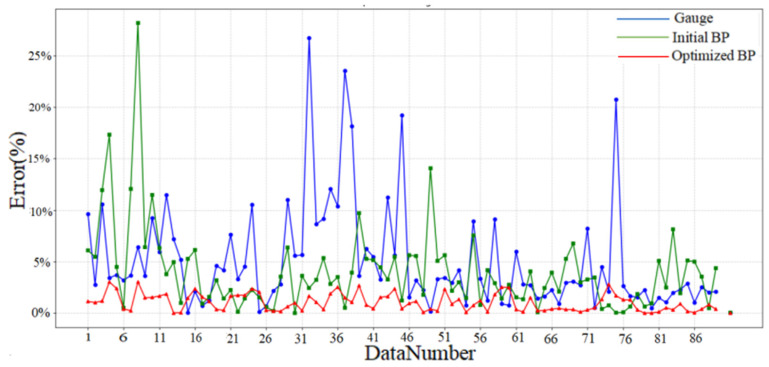
Error comparison: gauge vs. BP models.

**Figure 14:**
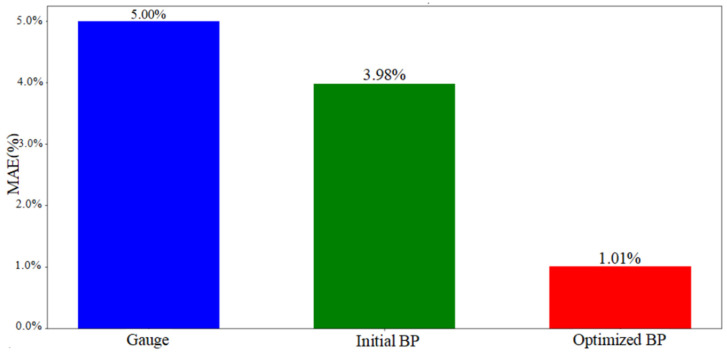
Mean absolute error comparison.

**Figure 15:**
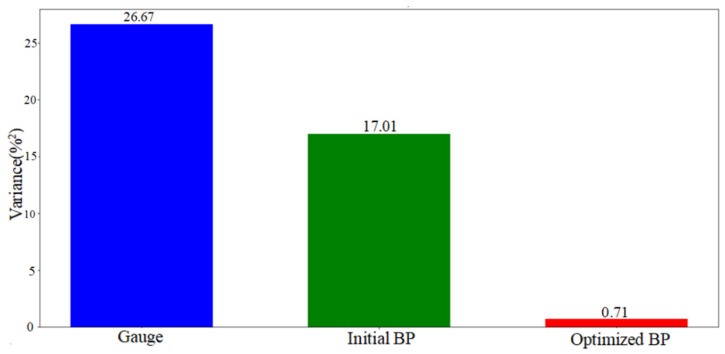
Error variance comparison.

To more thoroughly evaluate the neural network’s predictive performance, we computed and displayed diverse quantitative metrics. As illustrated in Figure 13, we analyzed the measurement errors of our 90 data points, along with the initial and optimized BP model prediction errors. The original measurement device showed higher error fluctuations than both BP models, while the optimized BP model demonstrated the lowest and most stable prediction errors. Additionally, we calculated the mean absolute error (MAE) (Figure 14) and error variance (Figure 15) between the measured, predicted, and actual values, presenting them in a bar chart (Figure 14). The original measurements exhibited the highest MAE and error variance, surpassing both BP models. The optimized BP model achieved the lowest prediction MAE of 1.01% and the smallest error variance of 0.71%^2^, highlighting its accuracy, stability, and superior performance.

## 5. Conclusions

This paper takes the Coriolis mass flow meter as the research object, primarily by constructing an experimental platform for coal–water slurry liquid–solid two-phase flow, collecting experimental data, and employing algorithm calibration methods. It investigates the errors generated by the Coriolis flow meter when measuring coal–water slurry liquid–solid two-phase flow under various influencing factors, as well as the causes of these related influencing factors. The paper focuses on developing a correction algorithm based on a genetic algorithm-optimized BP artificial neural network, achieving favorable results and providing a solution for future error correction research.

Compared with other papers, the innovations of this study are summarized as follows:

1. The first application of high solid content fluid measurement. This study introduces deep learning technology into the field of Coriolis flow meter error correction for high solid content coal–water slurry liquie–solid two-phase flow for the first time, breaking through the limitations of traditional methods in complex multiphase flow measurement and providing new ideas for high-precision industrial measurement.
2. Innovative design of hybrid model architecture. By integrating CNN (convolutional neural network) and LSTM (long short-term memory network) modules, the joint extraction of the spatiotemporal features of the multidimensional physical parameters of fluids (such as pressure difference, flow velocity, temperature, etc.) has been achieved. Further combining ResNet (residual network) and XGBoost (gradient boosting tree) optimization models significantly improved feature expression ability and regression accuracy, ultimately reducing the correction error from 3.98% in the initial model to 1.01%.
3. The combination of an experimental platform and a data-driven approach. The independently built liquid–solid two-phase flow experimental platform generated a measured dataset containing 90 sets of multidimensional parameters, providing a reliable foundation for the training and validation of deep learning models and solving the problem of data scarcity in this field.

Outlook:

We should explore more advanced deep learning architectures, such as transformers and graph neural networks, to more efficiently model spatiotemporal dependencies between multi-sensor data and enhance the adaptability to non-stationary flow fields. Research regarding lightweight model design (such as knowledge distillation and pruning techniques) should be pursued to reduce computational resource requirements and meet the deployment needs of industrial real-time monitoring scenarios.

## Figures and Tables

**Table 1:** Comparison of flow meter performance.

	Flowmeter	Coriolis Mass Flow Meter	Electromagnetic Flowmeter	Ultrasonic Flowmeter
Character				
Applicable Fluid		Gas/Liquid/Multi-Phase Flow	Conducting Flow	Clean or Contain Particulate Fluid
Type of Measurement		Direct Mass Flow	Volume Flow	Volume Flow Rate/Flow Velocity
Loss of Pressure		Secondary	None	None
Accuracy		±0.1–0.5%	±0.5–1%	±1–5%
Cost		High cost but low maintenance.	The cost is moderate, but the electrodes need to be cleaned.	High cost and affected by pipeline lining.

**Table 2:** Calibration results of the Dongfeng Electromechanical Z15 Coriolis Mass Flow Meter.

Flow Rate (t/h)	Weighed Value (Kg)	Displayed Value (Kg)	Relative Error (%)	Repeat- Ability (%)
350	3185.9	3181	1.5	0.46
	3173.8	3169	1.5	
	3224.7	3227	0.7	
300	3693.4	3701	2	0.89
	3689.9	3687	0.78	
	3726	3725	0.27	
250	3714.3	3703	3	0.95
	3346.7	3337	2.89	
	3400.5	3396	1.3	

**Table 3:** Experimental results of coal–water slurry solid–liquid two-phase flow.

Serial No.	Pressure Difference (MPa)	Flow Velocity (m/s)	Fluid Temperature (k)	Density (kg/m^3^)	Phase Difference (rad/s)	Time (s)	Weighed (g)	Displayed Value (g)	Error (%)
1	0.6	0.82	297.45	1196	0.425	149.35	1517.5	1667	9.9
2	0.61	0.97	297.45	1196	0.827	149.29	1514.2	1660	9.6
3	0.61	0.88	297.55	1196	0.562	149.29	1388.1	1426	2.7
4	0.61	1.18	297.85	1196	0.897	149.26	1321.5	1461	10.6
…									
90	0	3.67	314.45	1153	0.09	18.4	2121	2165	2.1

## Data Availability

The data presented in this paper are available by contacting the corresponding author.

## Abbreviations

BP	back propagation
CNN	convolutional neural network
CWS	coal–water slurry
CapsNet	capsule network
DBN-CapsNet	deep belief networks-capsule network
GBDT	gradient boosted decision tree
HIL	hardware-in-the-loop
IFD	incremental fault diagnosis
KD	knowledge distillation
KPCA	kernel principal component analysis
LSTM	long short-term memory
LASSO	least absolute shrinkage and selection operator
LAR	least angle regression
MEMAR	model evolution mechanism sample representative
QR	quantile regression
RBF	radial basis function
RCP	remote communication platform
ReLU	rectified linear unit
ResNet	residual networks
RNN	recurrent neural network
RTI	real-time interface
SVM	support vector machines
XGBoot	extreme gradient boosting
**List of Symbols**	
M	torque
F_C_	Coriolis force
m	particle mass
ω	angular velocity
V	radial velocity
θ	torsion angle
K	elastic coefficient
Q_m_	mass flow
